# Magic and mystery of microRNA‐32

**DOI:** 10.1111/jcmm.16861

**Published:** 2021-08-18

**Authors:** ZL Zeng, Qingyun Zhu, Zhibo Zhao, Xuyu Zu, Jianghua Liu

**Affiliations:** ^1^ The First Affiliated Hospital Department of Metabolism and Endocrinology Hengyang Medical School University of South China Hengyang China; ^2^ The First Affiliated Hospital Department of Clinical Medicine Hengyang Medical School University of South China Hengyang China; ^3^ Key Laboratory for Arteriosclerology of Hunan Province Department of Cardiovascular Disease Hengyang Medical School University of South China Hengyang China

**Keywords:** cancer, cardiovascular disorders, metabolic, microRNA

## Abstract

MicroRNAs (miRNAs) are a group of endogenous, small (∼22 nts in length) noncoding RNA molecules that function specifically by base pairing with the mRNA of genes and regulate gene expression at the post‐transcriptional level. Alterations in miR‐32 expression have been found in numerous diseases and shown to play a vital role in cell proliferation, apoptosis, oncogenesis, invasion, metastasis and drug resistance. MiR‐32 has been documented as an oncomiR in the majority of related studies but has been also verified as a tumour suppressor miRNA in conflicting reports. Moreover, it has a crucial role in metabolic and cardiovascular disorders. This review provides an in‐depth look into the most recent finding regarding miR‐32, which is involved in the expression, regulation and functions in different diseases, especially tumours. Additionally, this review outlines novel findings suggesting that miR‐32 may be useful as a noninvasive biomarker and as a targeted therapeutic in several diseases.

## 1 INTRODUCTION

**FIGURE 1:**
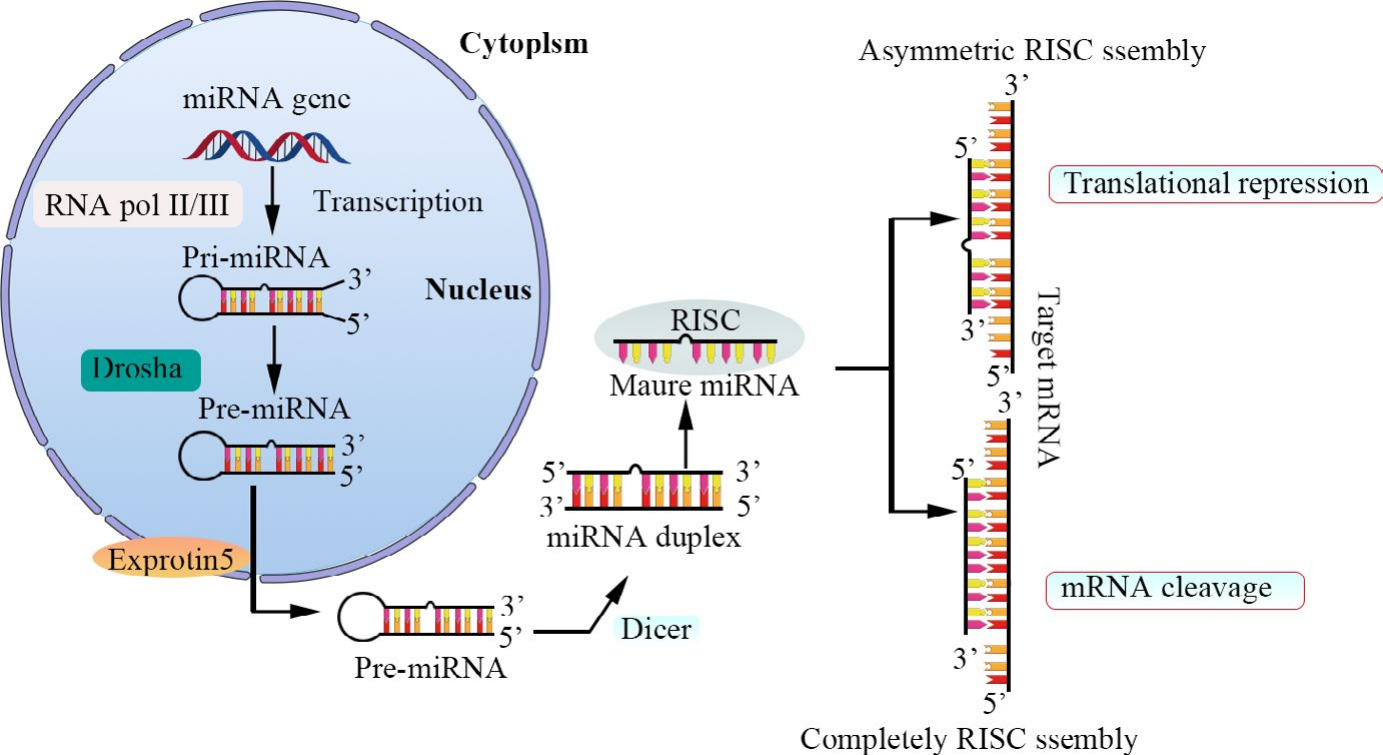
Schematic of miRNA biogenesis. The primary transcripts of miRNA genes (pri‐miRNAs) are transcribed by RNA polymerase II (pol II/III). The initiation step is mediated by the Drosha‐DGCR8 complex. The product of this nuclear processing step is an approximately 70‐nt precursor miRNA (pre‐miRNA). Then, pre‐miRNAs are transported from the nucleus to the cytoplasm through nuclear export factor exportin‐5. Once in the cytoplasm, pre‐miRNAs are recognized and processing step to produce miRNA duplexes by another RNaseIII, Dicer.Then, the duplexes are separated. One strand is usually selected as the mature miRNA and is loaded onto the RISC. If perfect complementarity with the 3′‐UTR of the target mRNAs, the target is cleaved and degraded, if not, the target is not cleaved, but translation is inhibited. In a few cases, miRNA can upregulate the transcription of the target mRNA

MicroRNAs (miRNAs) are endogenous, single‐stranded small noncoding RNAs about 19~24 nucleotides (nts) long that represent an emerging group of gene expression modulators with critical roles in several biological processes, such as cell proliferation, differentiation, cell cycle progression, autophagy, apoptosis and organ development. They are also used as noninvasive biomarkers and targeted therapeutics in several diseases, including atherosclerosis,^1^ tumours,^2^ diabetes^3^ and other diseases.^4^ Mature miRNA production occurs through a multistep process (Figure 1). MiRNAs can regulate up to 60% of protein‐coding genes at the post‐transcriptional level by forming the RNA‐induced silencing complex (RISC). Target transcripts are recognized by the RISC via direct binding to the 3'‐untranslated region (3'‐UTR) of the mRNA. miRNAs function through degradation of protein‐coding transcripts(perfect complementarity with the 3′‐UTR of the target mRNA) or translational repression(imperfect complementarity).^4^, ^5^

In 2017, our group constructed a miR‐32‐5p knockout mouse for the first time by using CRISPR/Cas9 technology (see Table 1 for the reference sequence) (Qing's master's thesis in Chinese). In the same year, we identified miR‐32‐5p as a potential key miRNA that is involved in vascular calcification (VC) in mice and humans. The mechanism of this involvement is as follows: miR‐32 modulates VC progression by activating phosphoinositide 3‐kinase (PI3K) signalling and increasing runt‐related transcription factor‐2 (RUNX2) expression and phosphorylation by targeting the 3′‐UTR of phosphatase and tensin homolog (PTEN) mRNA in vascular smooth muscle cells (VSMCs).^6^ In 2019, our group reported that miR‐32‐5p knockout eliminates lipopolysaccharide‐induced depressive‐like behaviour in mice through the inhibition of astrocyte overactivity.^7^ Thus far, our group has found that miR‐32 plays an important role in VC, atherosclerosis, diabetes, depression and inflammation (some data not shown).

**TABLE 1:** Reference sequence for miR‐32‐5p knockout in mice

sgRNA name	Oligo name	sgRNA sequence	Target
miR‐32‐5p‐sgRNA	Forward primier	caccggtactaagttgcatgttgtca	tactaagttgcatgttgtcacgg
	Reverse primer	aaactgacaacatgcaacttagtacc	

An increasing number of studies on miR‐32 have begun to focus on tumorigenesis and tumour progression in addition to vascular and metabolism‐related diseases mainly because miR‐32 regulates tumour cell apoptosis, proliferation and migration. MiR‐32 has been documented as an oncomiR in most studies, although conflicting reports have verified miR‐32 as a tumour suppressor miRNA. The contradictory role of miR‐32 in cancers may impede its application as a diagnostic and therapeutic target. Thus, exploring the possible mechanisms behind these contradictory findings is of great importance. We wrote this comprehensive review article to discuss in detail the role of miR‐32 in the pathogenesis and progression of vascular, metabolic and neoplastic diseases and the therapeutic potential of this molecule.

## 2 OVERVIEW OF MIR‐32

**FIGURE 2:**
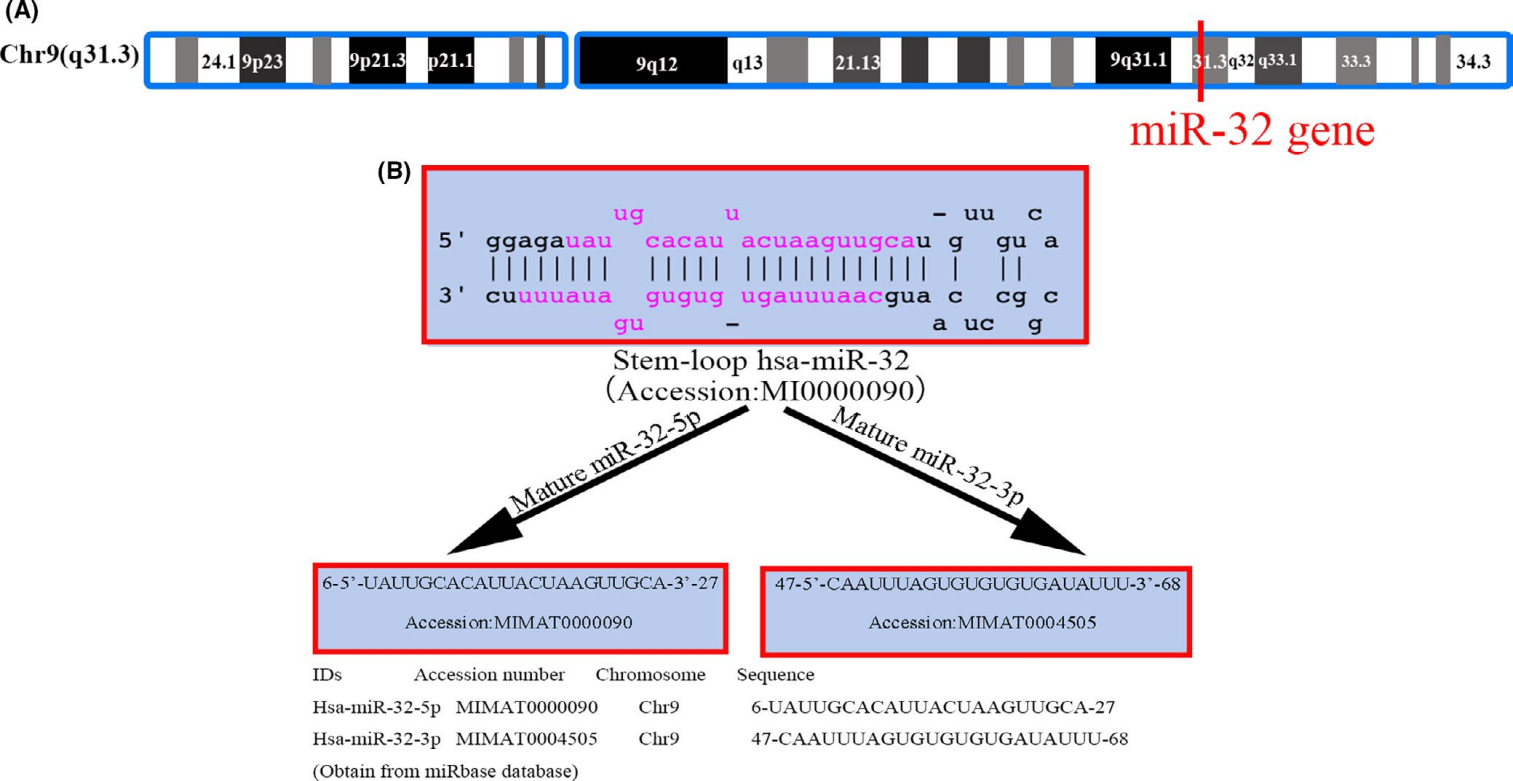
Location and sequence of miR‐32. (A) miR‐32 location. MiR‐32 is located on chromosome 9q31 (in the NR_029506.1 noncoding region); (B) miR‐32 sequence. The stem‐loop and the maturation sequences of miR‐32‐5p and miR‐32‐3p

MiR‐32 is an intronic miRNA that is located in intron 14 of c9orf5, the gene encoding transmembrane protein 245 (TMEM245), on chromosome 9.^8^ To our knowledge, human miR‐32 was first reported in 2001 by Dr. Thomas Tusachl's laboratory.^9^ According to miRBase, the family of miR‐32 includes 22 sequences. Among these sequences, hsa‐miR‐32‐3p and hsa‐miR‐32‐5p are located on chromosome 9 (chr9: 109 046 229–109 046 298, 9q31.3) and are highly conserved between species (according to miRcode, the miR‐32 gene is 89% and 61% conserved among primates and mammals, respectively).^10^ In further detail (Figure 2), miR‐32 is expressed in a variety of tissues in humans and mice, including serum,^11^ liver,^12^ kidney,^13^ breast^14^ and brain tissues.^7^ Wu et al.^15^ revealed the structure and regulation of the hsa‐miR‐32 promoter by using multimolecular biology methods, such as DNA pull‐down assay and mass spectrometry. Their results suggest that the core promoter region may be located within −320 bp to −1 bp of the 5′‐flanking region of the TMEM245/miR‐32 gene, whereas repressive regulatory elements may be located in the region from −606 bp to −320 bp. The transcription factors SMAD1, STAT1 and Foxk1 may be involved in the transcriptional regulation of miR‐32. As we will discuss in this review, miR‐32 has been identified to play a role in multiple diseases and the progression of diverse tumours, including colon cancer,^16^ oesophageal squamous cell carcinoma^17^ and gastric carcinoma.^18^

## 3 MIR‐32 IN THE CARDIOVASCULAR SYSTEM AND METABOLIC‐RELATED DISEASES

With the growing number of elders and obesity people, the incidence of cardiovascular and metabolic‐related diseases is increasing sharply, which greatly influence the morbidity and mortality of the general adults.^19^, ^20^ The role of miR‐32 in cardiovascular and metabolic‐related diseases is gradually being valued. Direct target genes that have been validated in various publications are displayed in Table 2. A gene must have been demonstrated to be directly affected by a miRNA on the basis of 3′ UTR luciferase reporter assay data to be considered as a target.

**TABLE 2:** Direct gene targets of miR‐32 in cardiovascular and metabolic‐related diseases

Disease	miR‐32 change	Target	Cell lines	In vivo verification	Target function	Reference
AMI	Up	KLF2	HUVECs	No	Suppress cell viability, pro‐inflammation	Dai et al. (2020)**Ref:22**
CCSCI	Down	NOTCH‐1	HUVECs	Yes	Inhibit angiogenesis	Cheng et al. (2020)**Ref:29**
VC	Up	PTEN	VSMCS	Yes	Inhibit VSMC osteogenic differentiation	Liu et al. (2017)**Ref:6**
DN	Up	SMAD7	HK‐2	Yes	Autophagy suppression, promote fibrosis,EMT and inflammation	Wang et al. (2020b)**Ref:13**
Cardiac fibrosis	Up	DUSP1	hCFs	No	Enhance apoptosis and induce the phenotypic alteration	Shen et al. (2019a)**Ref:38**

Abbreviations: AMI, acute myocardial infarction; CAC, coronary artery calcification; CCSCI, chronic compressive spinal cord injury; DN, Diabetic nephropathy; EMT, epithelial‐mesenchymal transition; hCFs, human cardiac fibroblasts; HUVECs, human umbilical vein endothelial cells; PTEN, phosphatase and tensin homolog; VC, vascular calcification; VSMCs, vascular smooth muscle cells.

### 3.1 Cardiovascular system

Cardiovascular diseases (CVDs), especially ischaemic heart disease, are the leading causes of deaths globally; approximately 17 million CVD‐caused deaths occur annually worldwide, and acute myocardial infarction (AMI)‐related mortality accounts for approximately 13% of these deaths.^21^ AMI is a serious CVD caused by coronary artery occlusion. Serum miR‐32‐5p expression is elevated in patients with AMI and shows a positive correlation with the biomarker levels of myocardial damage, endothelial injury and proinflammatory cytokines of AMI via target KLF2.^22^

Extracellular vehicles (EVs) including exosomes are nano‐sized lipid‐bound vesicles that are released from cells into the extracellular space.^23^ The expression levels of miR‐32‐5p were significantly higher in circulating exosomes from patient with stable coronary artery disease (SCAD) than those from the control group. The diagnosis AUC value is 0.691, which suggest that serum exosomal miR‐32‐5p may serve as potential diagnostic biomarkers for SCAD.^24^ Similar results were reported from another study, patient with CAD (All CAD patients were confirmed by angiographic evidence of >70% stenosis of at least 1 main coronary artery), plasma miR‐32‐3p was significantly higher than the control group, and the diagnosis AUC value is 0.745 (95%CI 0.649–0.84).^25^ All indicate that miR‐32 has promising diagnostic value for CAD.

Angiogenesis has both beneficial and deleterious effects.On the one hand, angiogenesis is beneficial for tissue growth and regeneration. On the downside, vessels can fuel inflammatory, malignant diseases and promote tumour metastasis. In addition, insufficient vessel growth or maintenance can lead to ischaemic disease like stroke, AMI, ulcerative disorders and neurodegeneration.^26^, ^27^, ^28^ X‐inactive specific transcript (Xist), a female‐specific long noncoding RNA (lncRNA), sponges miR‐32‐5p and modulates Notch‐1 expression. Xist promotes angiogenesis and microvessel density after chronic compressive spinal cord injury in vitro and in vivo.^29^

VC is a high‐incidence and high‐risk disease with increasing morbidity and high mortality.^30^ Observations from a registry of 25,253 patients show that coronary calcification (CAC) is an independent predictor of mortality. CAC is associated with a 12‐fold increased risk for hard coronary heart disease events.^31^ We previously found that the expression of miR‐32‐5p increases during the occurrence of VC in mice and humans. Furthermore, miR‐32‐5p promotes VSMC calcification by inducing the expression of VC markers via activating the PI3K‐Akt pathway by targeting PTEN and enhancing Runx2 expression and activity. Moreover, miR‐32 is upregulated in plasma from patients exhibiting CAC, indicating that miR‐32‐5p can be used as a potential CAC biomarker.^6^ Most recently, our group found that miR‐32‐5p promotes VSMCs calcification by upregulating TNFα in the microenvironment.^32^

### 3.2 Diabetes and its complications

The prevalence of type 2 diabetes mellitus (T2DM) increases in parallel with the ongoing global obesity epidemic.^33^ Diabetic nephropathy (DN) is a serious microvascular complication of diabetes, which is the primary cause of end‐stage renal disease (ESRD).^34^ MiR‐32‐5p is highly expressed in kidney tissue,^35^ and the higher expression of miR‐32 has been found in patients with obesity and T2DM and in streptozotocin (STZ)‐induced diabetic rats.^36^ In addition, high glucose (HG) greatly increases miR‐32 expression in HK‐2 cells. miR‐32 inhibition significant relieves HG‐mediated suppression of autophagy, fibrosis, epithelial‐mesenchymal transition (EMT) and inflammation. Moreover, miR‐32 overexpression magnificently reduction the expression of mothers against decapentaplegic homolog 7 (SMAD7), and the opposite effect was observed following knockdown of miR‐32. These results showed that miR‐32 may play roles in the progression of EMT and fibrosis in DN.^13^

Dual‐specificity protein phosphatase (DUSP) is known as a mitogen‐activated protein kinase (MAPK) phosphatase and is expressed at low levels in the myocardium of diabetic rats.^37^ High glucose levels result in miR‐32‐5p overexpression, which reduces the expression of DUSP1. The overexpression of miR‐32‐5p and the downregulation of DUSP1 promotes cell apoptosis and phenotypic changes in human cardiac fibroblasts, suggesting that miR‐32 also may play a role in diabetic‐related myocardial fibrosis.^38^

Approximately 70% of patients with T2DM have fatty liver disease and exhibit a course of liver fibrosis with increased severity.^39^ Growing evidence indicates that EMT serves a crucial role in the progression of liver fibrogenesis. MiR‐32 expression is markedly increased in the liver tissue of STZ‐induced diabetic rats and in AML12 cells under high glucose treatment and promotes liver fibrosis by directly targeting metastasis‑associated proteins.^12^

## 4 MIR‐32 IN CANCERS

**FIGURE 3:**
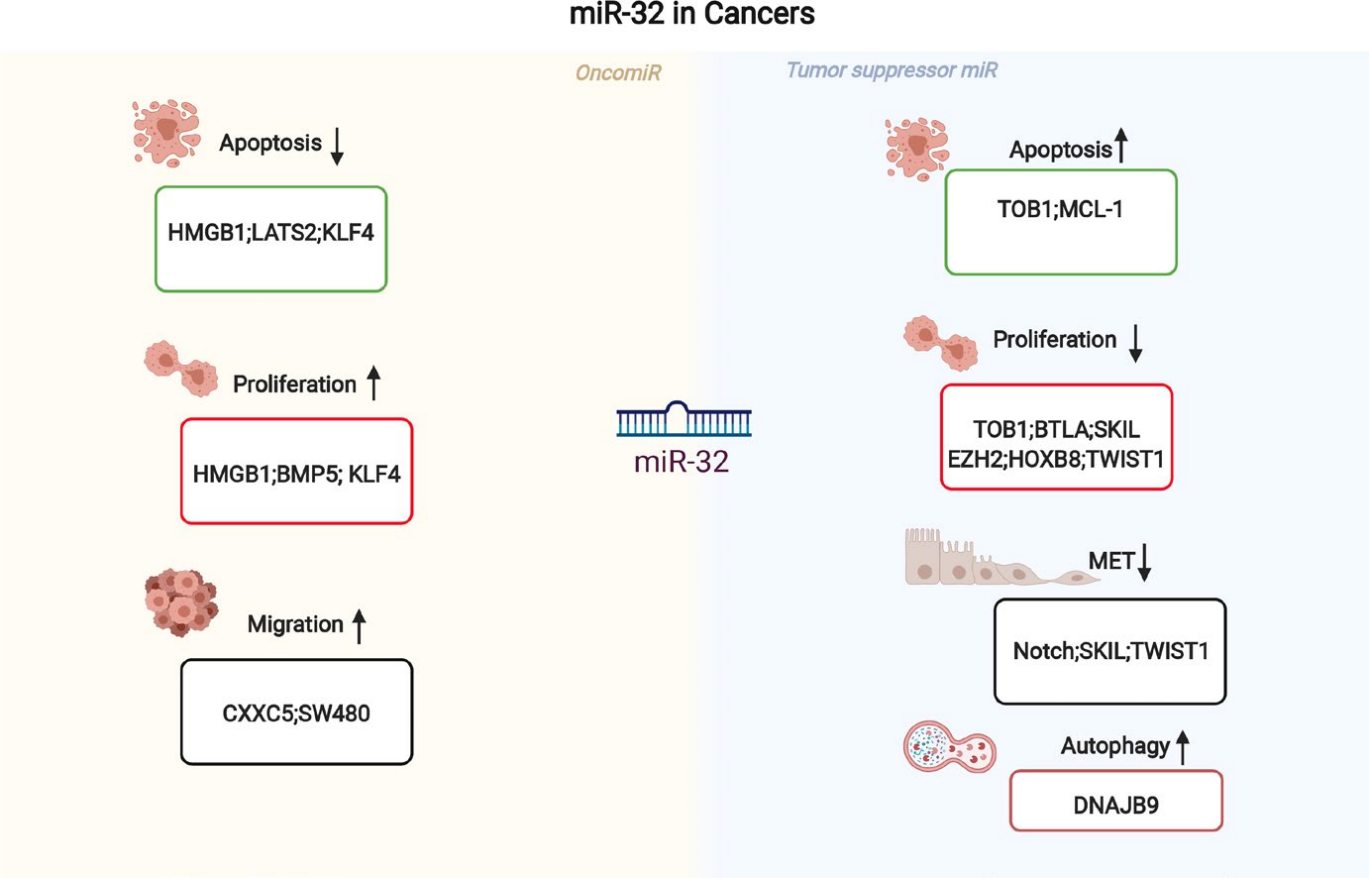
Role of miR‐32 as an oncomiR or as a tumour suppressor miRNA. The yellow background indicates the miR‐32 act as oncogenes, whereas the light blue background indicates the miR‐32 act as tumour suppressor genes. MiR‐32 is involved in the regulation of cell proliferation, migration, invasion, apoptosis and resistance to chemotherapeutic drugs by suppressing multiple targets

MiRNAs may act as oncogenes by targeting tumour suppressor genes or as tumour suppressors by either inhibiting cellular oncogene expression or regulating cell death (Figure 3). Moreover, the same miRNA may have different roles in various tumours.^40^ In the regulation of miRNA biogenesis, methylation and transcriptional changes in tumour tissues, the expression of some miRNAs changes accordingly partly due to the tumour microenvironment; these miRNAs may affect tumour prognosis by regulating genes related to tumorigenesis, regulating apoptosis, autophagy or affecting the tumour microenvironment and can thus be used as potential biomarkers.^41^, ^42^ MiR‐32 expression is changed in numerous cancer types. MiR‐32 has been identified as an oncomiR in the majority of research, but it has also been identified as a tumour suppressor miRNA in other findings. The molecular processes behind the alteration of miR‐32 in cancer have piqued the curiosity of researchers. Given that the same individual miRNAs can operate as tumour suppressors in certain cancer types and as oncomiRs in others, it is important to investigate their phenotypic effects and target genes independently in various malignancies. Table 3 shows direct target genes that have been validated in various cancers.

**TABLE 3:** Direct gene targets of miR‐32 in cancers

Disease	miR‐32 change	Target	Cell lines	In vivo verification	Target function	References
OS	Down	HMGB1	Five OS cell lines^a^	No	Induced apoptosis and impeded proliferation, migration, and invasion	Lou et al. (2020) Ref:52
Breast cancer	Down	TOB1	Human TNBC cell lines	No	Inhibits proliferation and induce apoptosis	Wang et al. (2020c) Ref:14
Retinoblastoma	Down	NOTCH	Retinoblastoma cell lines (Weri‐Rb1 and Y79)	Yes	Inhibit EMT	Gao et al. (2020)Ref:57
Ovarian cancer	Down	BTLA	Human SKOV3 and IOSE80 cells	No	Inhibits the proliferation, migration, and invasion	Zhang et al. (2020) Ref:60
AML	Down	DNAJB9	HL60 and HL60/ADR cells	No	Induce autophagy	Wang et al. (2020a)Ref:62
Myeloma	Up	PTEN	U266 cells	No	Enhance the proliferation and inhibits apoptotic	Sun et al. (2020b)Ref:67
CRC	Down	SKIL	LoVo, RKO, SW480, and HT‐29	Yes	Inhibits cell proliferation, metastasis, and EMT process	Ye et al. (2019)Ref:76
Glioma	Down	EZH2	U87, U251, A172, U118 and primary normal human astrocytes	No	Inhibits cell proliferation and metastasis	Chinaranagari et al. (2014)Ref:87
Nasopharyngeal carcinoma	Up	LATS2	C666‐1 (CC‐Y1082) and CNE2 (CC‐Y1119)	No	Inhibits apoptosis	Wang et al. (2019b)Ref:90
ESCC	Up	CXXC5	EC9706 and KYSE450	Yes	Promote migration, invasion, adhesion	Liu et al. (2019a)Ref:17
Colorectal cancer	Up	TOB1	SW480	No	Promote migration and invasion	Liang et al. (2019)Ref:69
Colorectal cancer	Up	BMP5	Lovo, HCT116, HT‐29	No	Promoted cell proliferation and migration	Chen et al. (2018)Ref:72
Cervical cancer	Down	HOXB8	siHa	No	Suppressed cell proliferation, invasion and migration	Liu et al. (2019b)Ref:94
PC	Up	KLF4	PC‐3, DU145, PANC‐1 and BxPC‐3	No	Inhibit cell apoptosis, promoted cell proliferation	Ref:100,103Gao et al. (2017) and Zhang et al. (2018)
Lung cancer	Down	TWIST1	H1299 and A549	Yes	Inhibits cell proliferation and EMT	Li and Wu (2016)Ref:85
Melanoma	Down	MCL‐1	ARF^−/−^,INK4a^−/−^ melanocytes and Primary melanocyte	Yes	Enhance apoptosis	Mishra et al. (2016)REF:10
Gastric cance	Up	KLF4	GES‐1, MGC8‐03, HGC‐27, NCI‐N87, AGS, SGC‐7901, MKN28, MKN‐45 and KATO‐III	No	Promotes cell proliferation, migration and invasion	Yan et al. (2015)REF:107

Abbreviations: AML, acute myeloid leukemia; CRC, colorectal cancer; ESCC, esophageal squamous cell carcinoma; OS, osteosarcoma; PBMCs, peripheral blood mononuclear cells; PC, prostate cancer.

^a^ Five OS cell lines: U2OS, Saos‐2, 143B, HOS, and MG63 and the hFOB1.19 cells.

### 4.1 Breast cancer

Breast cancer is the most common malignant tumour in women worldwide and is curable in ~70%–80% of patients with non‐metastasis breast cancer. However, advanced breast cancer with distant organ metastases is considered incurable with current strategies and agents.^43^ Triple‐negative breast cancer (TNBC) is a subtype of breast cancer with poor prognosis.^44^

LncRNAs are an extraordinary group of nonprotein‐coding RNAs that are longer than 200 nts in length but absence of protein‐coding potential.^45^ Certain lncRNAs can act as competing endogenous RNAs (ceRNAs), by competitively occupying the shared binding sequences of miRNAs, thus sequestering the miRNAs and altering the expression of their downstream target genes.^46^ Evidence showing that miR‐32 can interact with lncRNAs exists. LncRNA WEE2‐AS1 is often considered to be an oncogene.^47^ Interestingly, WEE2‐AS1 is also considered to be an atherosclerosis‐related gene. Antisense WEE2‑AS1 can regulate human vascular endothelial cell viability via the cell cycle G2/M transition in arteriosclerosis obliterans.^48^ Wang et al.^14^ reported that in TNBC cells, miR‐32‐5p is downregulated, whereas WEE2‐AS1 is upregulated. By acting as a sponge, WEE2‐AS1 can inhibit miR‐32‐5p expression. The WEE2‐AS1/miR‐32‐5p/TOB1 axis can negatively modulate cancer progression in TNBC cells by inhibiting the expression of TOB1, an oncogene.

### 4.2 Osteosarcoma

Osteosarcoma (OS) is a primary malignant tumour in children and adolescents.^49^ MiR‐32‐5p is present at decreased levels in OS tissues and cells. LncRNA HNF1A antisense RNA 1 (HNF1A‐AS1) is connected to the development of a range of cancers, such as hepatocellular cancer^50^ and OS.^51^ Moreover, HNF1A‐AS1 binds to miR‐32‐5p to regulate the expression of HMGB1‐induced cell apoptosis and impedes proliferation, migration and invasion in OS cells, indicating that HNF1A‐AS1 and miR‐32‐5p may be a potential biomarker and therapeutic target for the diagnosis and treatment of OS.^52^

### 4.3 Retinoblastoma

Retinoblastoma is a highly malignant tumour that appears in retinal development and is the most common primary intraocular tumour in childhood and infancy.^53^ Recent studies have shown that lncRNA‐ROR may contribute to the tumorigenesis and metastasis.^54^, ^55^, ^56^ LncRNA‐ROR is significantly upregulated in retinoblastoma tissues, and its overexpression is significantly correlated with optic nerve invasion, nodal or distant metastasis, and recurrence. LncRNA‐ROR modulates the EMT programme by competitively binding to endogenous miR‐32‐5p and regulating Notch signalling pathway activity in retinoblastoma cells; these behaviours may provide new insights into novel molecular therapeutic targets for retinoblastoma.^57^

### 4.4 Ovarian cancer

The incidence of ovarian cancer ranks sixth among female tumours. Meanwhile, its mortality ranks first among gynaecological tumours, with at least 120,000 deaths worldwide annually.^58^ Given its deep anatomical location, the early diagnosis of ovarian cancer is difficult. Nearly 75% of patients already exhibit pelvic metastasis or other distant metastasis upon diagnosis, and their 5‐year survival rate is only 30%.^59^ Zhang et al.^60^ reported that miR‐32 is significantly downregulated in ovarian cancer tissues and cells. The overexpression of miR‐32 significantly inhibits the proliferation, migration and invasion of ovarian cancer cells by regulating its target genes, namely, B and T lymphocytes attenuator (BTLA).

### 4.5 Haematological oncology

Acute myeloid leukaemia (AML) is an aggressive haematopoietic malignancy and the most common form of acute leukaemia in adults. Resistance to chemotherapy contributes to the poor outcome of AML. Although the application of new targeted therapies, multidrug combination chemotherapy and haematopoietic stem cell transplantation has greatly improved the prognosis of patients in recent years, an effective treatment for refractory and recurrent cases does not exist, and the prognosis of approximately 50% of patients remains poor due to chemotherapy resistance and recurrence.^61^

LncRNA SNHG5 is aberrantly overexpressed in AML relative to that in donors. SNHG5 functions as competitive RNA with miR‐32 to regulate DNAJB9 expression. SNHG5 increases chemotherapy resistance in AML cells by regulating autophagy via the miR32/DNAJB9 axis.^62^

T‐cell acute lymphoblastic leukaemia (T‐ALL) is an aggressive and malignant neoplasm that arises from haematopoietic T‐cell precursors.^63^ FBXW7 is a tumour suppressor gene in T‐ALL, which increases the stability of Notch‐1 protein and enhances the antitumour effect of p53, and the FBXW7 gene is frequently inactivated in T‐ALL.^64^ The expression levels of miR‐32 are significantly higher in patients with T‐ALL than in healthy individuals, and miR‐32 inhibits FBXW7 expression by targeting the 3′‐UTR of FBXW7; thus, miR‐32 and FBXW7 may become potential targets for the diagnosis and treatment of acute leukaemia.^65^

Myeloma remains an incurable plasma‐cell cancer.^66^ Sun et al.^67^ showed that in patients with myeloma, miR‐32, miR‐126, miR‐123 and miR‐183 are significantly highly expressed, whereas miR‐5, miR‐76 and miR‐50 are expressed at remarkably low levels. In myeloma cells, the overexpression of miR‐32 can significantly enhance proliferation capability and inhibit apoptosis by targeting PTEN, indicating the positive association between miR‐32 and myeloma.

### 4.6 Colorectal cancer

Colorectal cancer (CRC) is one of the most malignant cancers worldwide, which had caused several millions of deaths annually due to its late‐stage diagnosis, metastasis trend and high recurrence.^68^ The level of miR‐32‐5p is significantly increased in CRC tissues and positively correlated with tumour differentiation and metastasis. Log‐rank tests have shown that high levels of miR‐32‐5p are significantly correlated with poor overall survival and disease‐free survival. Moreover, the downregulation of miR‐32‐5p enhances radiosensitivity and inhibits migration and invasion by promoting TOB1 expression.^69^ TOB1, a member of the antiproliferative protein B‐cell translocation gene/transducer of the erbB2 family, can act as a tumour suppressor to inhibit cell proliferation, migration and invasion in different types of human cancers.^70^, ^71^

A PCR analysis of 28 pairs of CRC tissues and adjacent normal tissues has revealed that the expression of miR‐32 is significantly increased in CRC and that the overexpression of miR‐32 in LoVo cells promotes cell proliferation and migration through the direct targeting of tumour suppressor bone morphogenetic protein 5, whereas the inhibition of miR‐32 in HCT‐116 cells shows the opposite pattern.^72^

Another study reported the opposite result. SNHG14 serves as a tumour promoter that can facilitate breast cancer cell proliferation and invasion.^73^ Ski‐oncogene‐like (SKIL) also has been reported to exert an oncogenic impact on diverse cancers.^74^, ^75^ SNHG14 expression is increased in CRC cells.SNHG14 upregulation promotes metastasis and EMT. MiR‐32‐5p presents low expression, which is negatively regulated by SNHG14 via a ceRNA mechanism; furthermore, SKIL is a downstream target gene of miR‐32‐5p, and miR‐32‐5p downregulates SKIL expression by binding to the SKIL 3′UTR. In brief, SNHG14 regulates CRC progression via the miR‐32‐5p/SKIL axis, providing a novel point in the treatment of patients with CRC.^76^

Promoter methylation and other epigenetic events contribute to miRNA expression regulation in tumours.^77^ However, this regulation does not seem to affect the expression of miR‐32 in tumour cells. Bisulphate sequencing polymerase chain reaction (BSP) was used to analyse the effects of methylation on the expression of miR‑32 in the CRC cell lines HT‑29, HCT‑116 and the normal colonic epithelial cell line NCM460. The potential role of DNA methylation and histone acetylation on the regulation of miR‐32 gene expression in CRC cells was also investigated. BSP revealed that CpG sites in the miR‐32 promoter region of CRC and normal colonic epithelial cells are all hypomethylated with methylation rates of 0.12%, 1.14% and 0.64% in HCT‐116, HT‐29 and NCM460 cells, respectively. Treatment with 5‐Aza‐dC and/or TSA and transfection with DNMT1 plasmid does not significantly alter miR‐32 expression. This finding suggests that DNA methylation and histone acetylation have no effect on miR‐32 expression in CRC cells.^78^

### 4.7 Lung cancer

Lung cancer is one of the most common cancers that threaten human life and health worldwide. Non‐small cell lung cancer (NSCLC) is a heterogeneous class of tumours that accounts for approximately 85% of newly diagnosed lung cancer cases, and 70% of patients with NSCLC are at an advanced stage at the time of diagnosis.^79^ Many recent studies have demonstrated that EMT is one of the crucial molecular mechanisms inducing tumour invasion and metastasis.^79^, ^80^ Twist1 is a well‐known regulator of EMT, which suppresses E‐cadherin expression via transcriptional repression.^81^ MiRNA microarray studies have identified that the expression level of miR‐32 in NSCLC tissues is obviously decreased compared with that in nontumour tissues.^82^, ^83^ miR‐32 inhibits NSCLC cell growth,EMT,and metastasis by targeting TWIST1. Furthermore, an in vivo study confirmed that the overexpression of miR‐32 suppresses the growth of NSCLC tumours.^84^, ^85^ These data suggest that miR‐32 acts as a novel tumour suppressor in NSCLC pathogenesis.

Another study showed that the levels of miR‐32 have no significant difference in NSCLC patients pre‐and post‐treatment. However, plasma levels of miR‐32 were significantly higher after chemotherapy than those observed before chemotherapy. Moreover, high miR‐32 levels are associated with improved chemotherapy efficacy. Thus, changes in plasma miR‐32 levels are prognostic indicators for lung cancer patients receiving platinum‐based chemotherapy.^86^

### 4.8 Glioma

Enhancer of zeste homolog 2 (EZH2) is the catalytic subunit of the polycomb repressive complex 2 (PRC2), which participates in cell cycle regulation and carcinogenesis through methylating H3K27.^87^ Zhang et al.^88^ reported that miR‐32 is downregulated in glioma tissues and cells and has an important role in inhibiting glioma cell proliferation and metastasis by suppressing the expression of EZH2 by directly targeting its 3′‐UTR.

### 4.9 Nasopharyngeal carcinoma

Nasopharyngeal carcinoma (NPC) is a highly malignant epithelial carcinoma arising from the epithelial lining of the nasopharynx.^89^ Several recent studies have explored the molecular mechanism of nasopharyngeal carcinoma. The expression of miR‐32 is upregulated in nasopharyngeal carcinoma tissues. By downregulating miR‐32, isoliquiritigenin promotes nasopharyngeal carcinoma cell apoptosis by the upregulation of proapoptotic genes, including Bax, caspase 9 and caspase‐3, and downregulation of the antiapoptotic marker genes Bcl‐2. Meanwhile, isoliquiritigenin suppresses nasopharyngeal carcinoma cell migration and invasion with the downregulation of matrix metalloproteinase (MMP)‐2 and MMP‐9. This effect may be related to the capability of isoliquiritigen to increase the expression of large tumour suppressor 2, which is the target of miR‐32.^90^

### 4.10 Oesophageal squamous cell carcinoma

Oesophageal squamous cell carcinoma (ESCC) is recognized as a malignant tumour with poor prognosis.^91^ The expression of miR‐32 in ESCC tissues and cells is significantly increased. Downregulation of miR‐32 inhibits ESCC Cell proliferation, migration and invasion. In vivo, miR‐32 inhibitors decrease tumour size, weight and metastatic nodule number. Their biological effects may be attributed to the inhibition of TGF‐β signalling mediated via the targeting of the 3′‐UTR of CXXC5.^17^

### 4.11 Cervical cancer

Cervical cancer (CCa) is one of the most common female cancers globally.^92^ The World Health Organization states that approximately 530,000 patients with CCa are diagnosed every year around the world; this number progressively increases annually at a rate of 5% of the total female population and skews young.^93^ Liu et al.^94^ posted data showing that in CCa tissue and cell lines, miR‐32‐5p is expressed at significantly reduced levels and can inhibit cellular malignant behaviour by regulating the expression of HOXB8.

### 4.12 Prostate cancer

Prostate cancer (PC) is the second most common urological malignancy and the sixth leading cause of cancer‐associated mortality in males worldwide.^95^ Early diagnosis and treatment are crucial because the localized PC can be cured by the radical prostatectomy or definitive radiation therapy. However, castration‐resistant PC, an advanced form of the disease, lacks curative treatment.^96^ Prostate‐specific antigen (PSA) screening for prostate cancer in men of average risk remains controversial. Moreover, the use of PSA also is associated with overdiagnosis.^97^ Therefore, more specific biomarkers are required for PC diagnosis and prognosis, as well as for therapeutic targets. Numerous studies have demonstrated miRNAs are potential markers for the diagnosis, prognosis, classification, staging and therapeutic monitoring of cancers. The elevated expression of miR‐32 increases proliferation and decreases PC cells apoptosis.^8^, ^98^ Leena et al.^99^ showed that transgenic miR‐32 expression increases replicative activity and promotes metaplastic transformation in the mouse prostate epithelium and identified RAC2 as a potential and clinically relevant target of miR‐32. Therefore, miR‐32 is considered as a potential biomarker for the diagnosis and prognosis of PC.

Chemotherapeutic insensitivity remains a massive challenge in PC treatment. The downregulation of miR‐32‐5p by cisplatin induces the expression of KLF4 by directly binding to the promoter of BIK, facilitating its transcription and promoting prostate cell apoptosis; these events result in an increase in the chemosensitivity of PC.^100^

LncRNA growth arrest‐specific transcript 5 (GAS5) is a well‐known tumour suppressor gene in several human cancers.^101^, ^102^ Gao et al.^103^ found that GAS5 is decreased but miR‑32‑5p is increased in human PC tissues and cells from 22 patient samples. GAS5 negatively regulates miR‐32‐5p expression, which promotes the expression of PTEN, a well‐known tumour suppressor. PTEN can block PI3K/Akt signalling pathway activation, thus resulting in the inhibition of the proliferation and survival of PC cells. Therefore, GAS5 suppresses the proliferation, migration and invasion of PC cells partially under the mediation of the miR‐32‐5p/PTEN axis.

### 4.13 Melanoma

Cutaneous malignant melanoma is among the deadliest human cancers that is broadly resistant to most clinical therapies.^104^ MiR‐32 is downregulated in primary and metastatic melanoma, and the overexpression of miR‐32 induced apoptosis and reducing anchorage‐independent growth in melanoma cells via downregulates pMEK levels by targeting MCL‐1 3′UTR. Furthermore, the efficacy of miR‐32 expression in inhibiting tumour growth in vivo has been validated. This tumour growth inhibitory effect of miR‐32 is more effective than that of vemurafenib, a BRAFV600E inhibitor. Moreover, the combination of miR‐32 and vemurafenib is more effective than either that of vemurafenib or miR‐32 treatment alone, therefore suggesting that miR‐32 acts as a tumour suppressor in melanoma cells.^10^

### 4.14 Gastric cancer

Gastric cancer (GC) is a high‐incidence malignant tumour with a poor prognosis that poses a serious threat to global health. The International Agency for Research on Cancer reported approximately 951,000 newly diagnosed cases of gastric cancer worldwide and 723,000 related deaths in 2012. Among all cancers, GC ranks the fourth and the fifth respectively among males and females worldwide in terms of incidence rate, while it ranks the third and the fifth respectively in terms of mortality rate.^105^ Serum miR‐32‐5p expression was higher in GC patients than that in healthy controls.

MiR‐32 acts as an oncogene by directly targeting KLF4, a member of the KLF family of transcription factors, which acts as tumour suppressor in certain cancers, including GC, by regulating proliferation, differentiation, apoptosis and somatic cell reprogramming. The knockdown of KLF4 can mimic the effect of miR‐32 overexpression on cell proliferation, invasion and metastasis. The levels of KLF4 mRNA in 43 clinical gastric carcinoma tissue and their adjacent normal tissue samples from the same patient with miR‐32 expression were examined by using qPCR. The results showed that KLF4 mRNA is expressed at significantly lower levels in GC than in adjacent tissues. Moreover, the authors identified a significant inverse correlation between miR‐32 and KLF4 in GC,^106^, ^107^ suggesting that the miR‐32‐KLF4 axis may be useful targets for GC diagnosis and therapeutics.

### 4.15 Clear cell renal cell carcinoma

Although clear‐cell renal cell carcinoma (ccRCC) is the most common histologic subtype of renal cell carcinoma and accounts for 70% of the cases of this malignancy, its detailed metastasis mechanisms remain unclear.^108^ NR2C2 (nuclear receptor subfamily 2, group C, member 2), also known as testicular orphan nuclear receptor 4 (TR4), is a transcription factor and a member of the nuclear receptor family.^109^ It may play positive roles in ccRCC metastasis as reflected by its higher expression in ccRCC tumours from patients with distant metastases than patients without distant metastases. In vitro studies involving multiple ccRCC cell lines (ACHN, OSRC‐2 and SW839 cell lines) also confirmed TR4’s positive role in promoting ccRCC cell invasion/migration. Mechanism dissection revealed that miR‐32‐5p can suppress TR4 by directly binding to the 3'UTR of TR4 mRNA, and TR4 may then alter HGF/Met signalling at the transcriptional regulation level by binding directly to the TR4 response elements on the HGF promoter.^110^

## 5 CONCLUSION AND PERSPECTIVES

**FIGURE 4:**
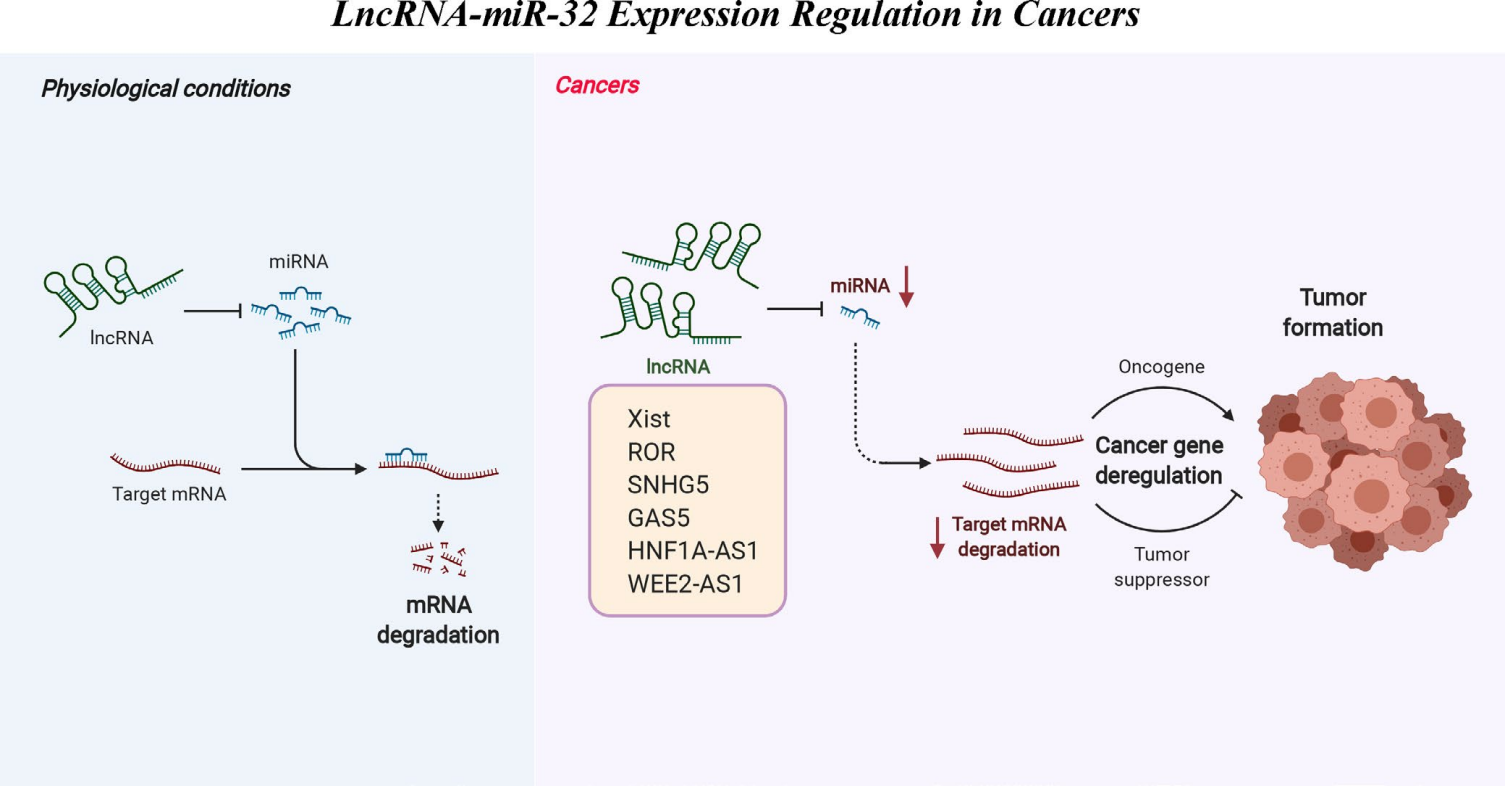
Gene expression regulation by lncRNA‐miR‐32 in cancers. During tumour occurrence, the expression levels of a series of lncRNAs been changed, which act as competing endogenous RNAs regulation miR‐32 expression, thus leading to cancer gene deregulation. This phenomenon ultimately promotes the development of tumours

In the past decade, we and other groups have found through deep and extensive studies that miR‐32 has a broad regulatory role in biological events, especially in tumorigenesis and cardiovascular system. The upstream of miR‐32 is mainly regulated by a series of lncRNAs that act as competing endogenous RNAs (Figure 4). The expression of miR‐32 may be upregulated or downregulated in different cancers, which may act as a tumour suppressor miR or oncomiR. The opposite expression trends and effects of miR‐32 have been reported even in the same cancer. The roles of miR‐32 in different cancers, as suggested by the literature, are shown in Table 3. Moreover, as shown in Table 4, we have summarized the research data based on clinical samples. The contradictory role of miR‐32 in cancers may impede its application as a diagnostic and therapeutic target, and exploring the possible mechanisms behind these contradictory findings is of great importance.

**TABLE 4:** Clinical studies investigating miR‐32 in patients

Sample size	Origin	Disease	miR‐32 change	References
88	Serum	AMI	Up	REF:22
66	Serum	CAC	Up	Liu et al. (2017)REF:6
68	OS tissues	Osteosarcoma	Down	Lou et al. (2020)REF:52
58	Retinoblastoma and adjacent non‐tumor tissues	Retinoblastoma	Down	Gao et al. (2020)REF:57
100	Ovarian cancer tissues and adjacent normal tissues	Ovarian cancer	Down	Zhang et al. (2020)REF:60
34	PBMCs	AML	Down	Wang et al. (2020a)REF:62
80	Peripheral blood	ALL	Up	Mansouri et al. (2020)REF:65
29	Myeloid tissues	Myeloma	Up	Sun et al. (2020b)REF:67
60	ESCC tissues	ESCC	Up	Liu et al. (2019a)REF:17
54	Colorectal cancer tissues	Colorectal cancer	Up	Liang et al. (2019)REF:69
28	Colorectal cancer tissues and adjacent normal tissues	Colorectal cancer	Up	Chen et al. (2018)REF:72
80	CCa tissues and adjacent normal tissues	Cervical cancer	Down	Liu et al. (2019b)REF:94
22	PC tissues and the adjacent normal tissues	Pancreatic cancer	Up	Gao et al. (2017)REF:103

Abbreviations: ALL, acute lymphoblastic leukemia; AMI, acute myocardial infarction; AML, acute myeloid leukemia; CAC, coronary artery calcification; ESCC, esophageal squamous cell carcinoma; OS, osteosarcoma; PBMCs, peripheral blood mononuclear cells.

Considering the considerable effects of miR‐32 on cell proliferation and survival, miR‐32 may be involved in a variety of pathophysiological processes, such as atherosclerosis, diabetes, ageing and tumours. Great space and value for exploration in these areas remain. Moreover, recent studies have found that another important function of microRNA is to act as a communication medium among cells and organs.^111^ MicroRNAs encased in extracellular vesicles (especially, exosomes) for long‐distance transport can regulate the function of adjacent and even long‐range cells, tissues or organs because exosomes/extracellular vesicles can encapsulate microRNAs to reduce degradation, thus increasing the likelihood of their action as diagnostic and prognostic biomarkers. This effect has become a promising research direction.

## CONFLICT OF INTEREST

The authors declare that they have no known competing financial interests or personal relationships that could have appeared to influence the work reported in this paper.

## AUTHOR CONTRIBUTIONS

**ZL Zeng:** Data curation; Software; Visualization; Writing‐original draft; Writing‐review & editing. **Qingyun Zhu** **:** Data curation; Writing‐original draft. **Zhibo Zhao:** Data curation; Writing‐review & editing . **Xuyu Zu:** Conceptualization Supervision . **Jianghua Liu:** Conceptualization; Supervision.
